# Validity and Reliability of a Smartphone App for Gait and Balance Assessment

**DOI:** 10.3390/s22010124

**Published:** 2021-12-25

**Authors:** Usman Rashid, David Barbado, Sharon Olsen, Gemma Alder, Jose L. L. Elvira, Sue Lord, Imran Khan Niazi, Denise Taylor

**Affiliations:** 1 Health & Rehabilitation Research Institute, Auckland University of Technology, Auckland 0627, New Zealand; sharon.olsen@aut.ac.nz (S.O.); gemma.alder@aut.ac.nz (G.A.); sue.lord@aut.ac.nz (S.L.); imran.niazi@aut.ac.nz (I.K.N.); denise.taylor@aut.ac.nz (D.T.); 2 Department of Sport Science, Sports Research Centre, Miguel Hernandez University of Elche, 03202 Elche, Spain; dbarbado@umh.es (D.B.); jose.lopeze@umh.es (J.L.L.E.); 3 Institute for Health and Biomedical Research (ISABIAL Foundation), Miguel Hernandez University, 03550 Alicante, Spain; 4 Centre for Chiropractic Research, New Zealand College of Chiropractic, Auckland 1060, New Zealand; 5 Centre for Sensory-Motor Interaction (SMI), Department of Health Science and Technology, Aalborg University, 9220 Aalborg, Denmark

**Keywords:** smartphones, app, gait, balance, validity, reliability

## Abstract

Advances in technology provide an opportunity to enhance the accuracy of gait and balance assessment, improving the diagnosis and rehabilitation processes for people with acute or chronic health conditions. This study investigated the validity and reliability of a smartphone-based application to measure postural stability and spatiotemporal aspects of gait during four static balance and two gait tasks. Thirty healthy participants (aged 20–69 years) performed the following tasks: (1) standing on a firm surface with eyes opened, (2) standing on a firm surface with eyes closed, (3) standing on a compliant surface with eyes open, (4) standing on a compliant surface with eyes closed, (5) walking in a straight line, and (6) walking in a straight line while turning their head from side to side. During these tasks, the app quantified the participants’ postural stability and spatiotemporal gait parameters. The concurrent validity of the smartphone app with respect to a 3D motion capture system was evaluated using partial Pearson’s correlations (r_p_) and limits of the agreement (LoA%). The within-session test–retest reliability over three repeated measures was assessed with the intraclass correlation coefficient (ICC) and the standard error of measurement (SEM). One-way repeated measures analyses of variance (ANOVAs) were used to evaluate responsiveness to differences across tasks and repetitions. Periodicity index, step length, step time, and walking speed during the gait tasks and postural stability outcomes during the static tasks showed moderate-to-excellent validity (0.55 ≤ r_p_ ≤ 0.98; 3% ≤ LoA% ≤ 12%) and reliability scores (0.52 ≤ ICC ≤ 0.92; 1% ≤ SEM% ≤ 6%) when the repetition effect was removed. Conversely, step variability and asymmetry parameters during both gait tasks generally showed poor validity and reliability except step length asymmetry, which showed moderate reliability (0.53 ≤ ICC ≤ 0.62) in both tasks when the repetition effect was removed. Postural stability and spatiotemporal gait parameters were found responsive (*p* < 0.05) to differences across tasks and test repetitions. Along with sound clinical judgement, the app can potentially be used in clinical practice to detect gait and balance impairments and track the effectiveness of rehabilitation programs. Further evaluation and refinement of the app in people with significant gait and balance deficits is needed.

## 1. Introduction

Balance and gait impairments profoundly impact mobility-related activities of daily living [1,2]. These impairments increase the risk of falling [3] and are associated with a reduced quality of life [4]. Accurate assessments of balance and gait are essential for the rehabilitation of many conditions, such as vestibular disorders [5], multiple sclerosis [6,7], Parkinson’s disease [8], stroke [9], and assessing the risk of falls in older adults [10]. Standardised scales of balance and gait are commonly used in clinical practice, including the Berg Balance Scale, the Functional Gait Assessment, and the 10 m walk test [11,12,13]. However, these scales depend on the ability of the clinician to observe and categorize an individual’s motor behaviour under broad performance categories and therefore have a limited ability to evaluate specific impairments accurately. In addition, these standardized clinical scales are prone to ceiling effects [14,15,16], which limit their ability to discriminate between different balance levels at their higher-end, especially for individuals with high scores at the onset of rehabilitation. These constraints can reduce the responsiveness of the clinical scales to small changes that occur during rehabilitation or as a result of a change in health condition [17,18].

Gold standard gait and balance measures can be found in the laboratory setting (for example, force platforms, 3D motion capture systems, inertial sensors) but are not routinely available to clinicians owing to their cost and the need for a skilled operator. Ongoing advances in inertial sensor technology have led to accelerometers and gyroscopes embedded in common electronic devices such as smartphones and iPods [19,20]. These sensors are low cost, portable, and easy to use, facilitating balance and gait evaluation in real-world ecologically valid settings. Inertial sensors embedded in electronic devices have been shown to reliably quantify postural sway in different static and dynamic conditions [21,22,23,24] and to reliably quantify spatiotemporal gait parameters [25,26]. Smartphone apps that evaluate gait or balance are available [24,27,28,29,30,31,32,33,34,35], although none evaluate both static and dynamic balance, both of which are key to safe and effective mobility. Here, we introduce a new smartphone app (the Gait&Balance app) that uses a structured and efficient protocol to evaluate performance during static balance and dynamic gait tasks that are commonly used in clinical practice. With the use of sensors embedded in standard smartphones, this technology has the potential to provide clinicians and sports professionals with easy access to more accurate and sensitive measures of balance and gait. This enhanced ability to assess and monitor progress may facilitate clinical decision making and optimize rehabilitation.

This study tested concurrent validity, test–retest reliability, and responsiveness of the smartphone-based application to measure performance during four static balance tasks and two gait tasks. The gold standard measure for concurrent validity was a 3D motion capture (MoCap) system.

## 2. Methods

### 2.1. Participants

Thirty healthy participants (15 females) were recruited in this study (median age: 46 ± IQR 27, min-max: 25–69 years; median height 167 ± IQR 17, min-max: 145–186 cm; median mass 76 ± IQR 19, min-max: 60–104 kg). Sex, age, height, and mass of each participant are listed in Table 1. Three females and three males were recruited in each 10-year age band from 20 to 69 years. Participants reported they had no musculoskeletal, neurological, or medical condition that may impair balance or limit their ability to perform the balance and gait tasks. At study entry, participants gave written informed consent in accordance with the Declaration of Helsinki. All experimental procedures were approved by the ethics committee of Miguel Hernandez University (DPS.FVG.01.18).

### 2.2. The Gait&Balance App

**Figure 1:**
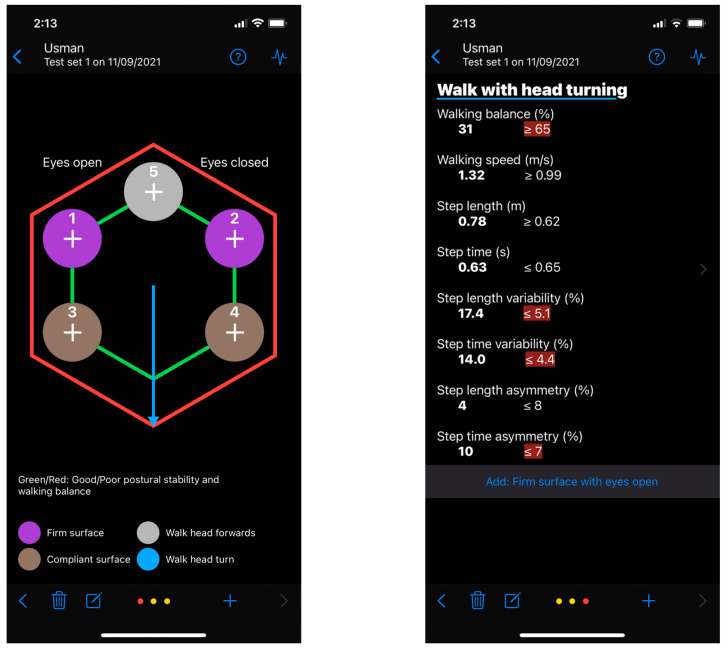
Screenshot images of the current version of the Gait&Balance application. Note: These screenshots were taken on the current beta version (0.3.4) and the app may change substantially in a future release.

The Gait&Balance (G&B) app is a smartphone application that analyses gait and balance through the inertial sensors embedded within the smartphone. G&B includes six gait and balance assessment tasks chosen from standardized observational clinical tools [11,12,13]. The static balance tasks included in the G&B app evaluate postural sway during the manipulation of sensory inputs required for balance [36,37]. There are four static test conditions. Users are required to stand as still as possible on a: (1) firm surface with eyes open (FS_EO_), (2) firm surface with eyes closed (absent visual information) (FS_EC_), (3) compliant surface with eyes open (altered proprioceptive feedback) (CS_EO_), and (4) compliant surface with eyes closed (absent visual information and altered proprioceptive information) (CS_EC_). Each task is performed for up to 30 s. The app provides “ready, set, go” and “rest” auditory cues at the start and end of each task. The final two tasks are performed during gait. Users are required to: (5) walk in a straight line facing forwards (WT_HF_), and (6) walk in a straight line while they turn their head from side to side (WT_HT_). The gait tasks are designed to be performed at the user’s preferred walking speed. Each gait task consists of four walks of 6 s duration. Each 6-s walk starts with a “ready, set, go” auditory cue and finishes with a “rest, turn around” cue. This task design allows the gait assessment to be carried out over approximately 10 m. Two screenshot images of the current version of the application are shown in Figure 1. A reasonable request to obtain the app can be made by contacting the corresponding author.

### 2.3. Experimental Procedure

Participants wore a sacroiliac belt (Posture Magic Sacroiliac SI Joint Support Belt) [38], which had been modified to firmly house an iPhone 7 (Apple, Inc., Cupertino, CA, USA) over the lower back (around L5/S1). Participants performed the six G&B app tasks three times, with a rest period of 30 s between tasks and 2 min between each set of six tasks. The six tasks were performed in the following order: (1) FS_EO_, (2) FS_EC_, (3) CS_EO_, (4) CS_EC_, (5) WT_HF_, (6) WT_HT_. A medium density foam mat (52 Kg/m^3^, 50 × 28 × 5 cm, Elksport, Zaragoza, Spain) was used as the compliant surface. Participants were asked to stand as still as possible with their feet hip-width apart and arms by their sides [39,40]. Any balance task was stopped if a participant could not maintain their position or lifted their arms or opened their eyes during the eyes-closed conditions. During the tasks, data from the smartphone inertial sensors (accelerometer and gyroscope) were recorded by the G&B app at 100 samples/s. Body kinematics were recorded through the Vicon 3D motion capture system (Vicon MX, Oxford, UK) at 200 samples/s using seven T10 cameras and five passive retro-reflective markers. One marker was placed on the centre of the smartphone, and two were placed on each foot, at the posterior calcaneus and lateral fifth metatarsal. The displacement signals were captured with respect to a frame of reference affixed to the lab. Data were reconstructed using Nexus 2.1. software (Vicon MX. Oxford, UK).

### 2.4. Data Processing

**Figure 2:**
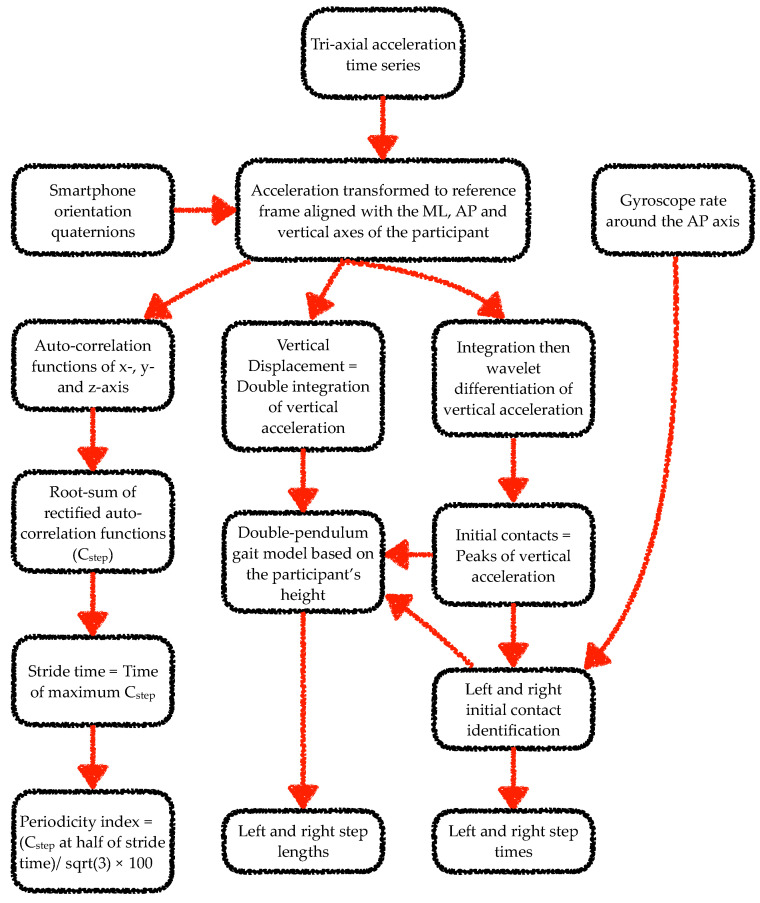
Flow chart of the steps involved in processing of smartphone data from each 6-s walking trial of the gait tasks. Note: ML and AP stand for mediolateral and anterior–posterior, respectively.

**Figure 3:**
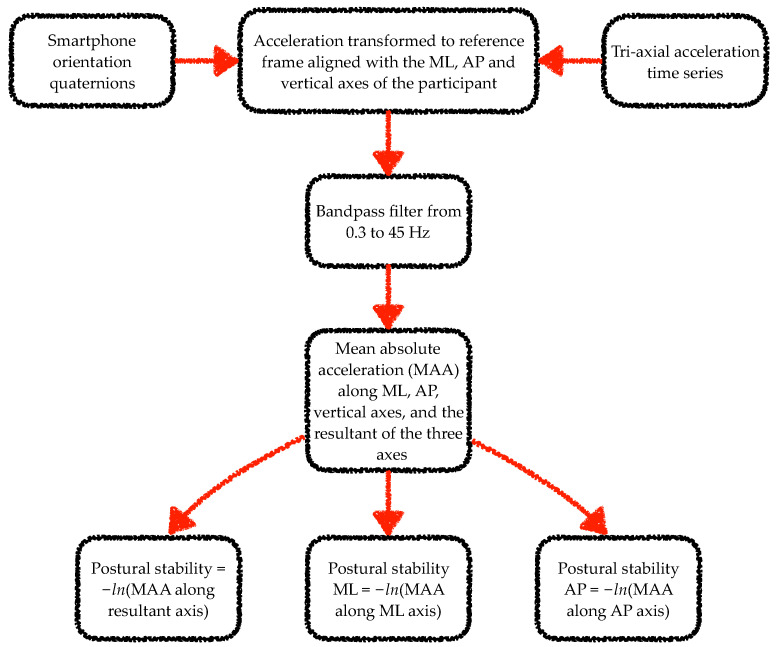
Flow chart of the steps involved in processing of smartphone data from the static balance tasks. Note: ML and AP stand for mediolateral and anterior–posterior, respectively. *ln* stands for natural logarithm.

The data from the smartphone inertial sensors were analysed using algorithms adapted from previous works [25,26,41,42,43,44,45]. Specifically, a wavelet-based step-event detection algorithm and a double-pendulum gait model were used to analyse gait data. From each walk, the gait analysis algorithm excluded data corresponding to the first stride. For the static balance task analysis, mean absolute acceleration time series were computed from the tri-axial acceleration time series [46,47]. Figure 2 and Figure 3 illustrate the steps involved in the processing of the smartphone data from the gait and static balance tasks, respectively. For the 3D MoCap data during the gait tasks, the whole 6-s walk was discarded if the calcaneus or fifth metatarsal marker displacement trajectories were missing for more than 10% of the gait segment. If data capture was below 10% the missing samples were filled with a shape-preserving piecewise cubic spline interpolation algorithm. Initial contact of the foot to the ground was identified from the calcaneus markers’ vertical velocity using a modified version of the foot velocity algorithm [48]. The first two initial contacts (one left and one right step) were discarded for the sake of similarity with the app’s gait analysis algorithm. For the 3D MoCap data during the static balance tasks, the raw kinematic data were transformed from the lab reference frame to each participant’s reference frame. In the participant’s reference frame, the kinematic time series in the x-, y- and z-axes corresponded to the mediolateral (ML), anterior–posterior (AP), and vertical axes.

### 2.5. Gait and Balance Outcomes

Gait and balance outcomes were based on past studies involving gait and balance assessment using a single body-worn inertial sensor [46,49]. The gait outcomes covered five dimensions of gait, namely, pace, rhythm, variability, asymmetry, and postural control [49]. These outcomes were:

(a) Periodicity index (also known as gait symmetry index [44]; units: %)

This parameter was computed from the root-sum of rectified auto-correlation functions of the tri-axial acceleration signals (C_step_) at half stride time. Stride time was computed by dividing the index of the maximum value of C_step_ by the sample rate. Periodicity was quantified as a percentage of the maximum possible value of C_step_ ($i.e.,\;\sqrt{3}$). Low periodicity scores may indicate step asymmetry and/or a high variability across strides. For the 3D MoCap system, the displacement signal was first numerically differentiated twice to obtain acceleration. A wavelet-based differentiation algorithm was used to avoid the amplification of high-frequency noise caused by numerical differentiation [50]. Periodicity was calculated for each 6-s walking trial, and its mean value across the trials was estimated by taking the median of the four individual trial values. Median was used as the best estimator for mean in the presence of data skew resulting from a potential algorithm or signal anomaly [51]. This outcome encompassed the step symmetry between the right and left step within a stride and the gait regularity across strides.

(b) Average step length (SL_Av_, units: m)

This parameter was computed as the mean of the AP distance between two consecutive initial contacts of alternative feet. For the 3D MoCap system, step lengths were calculated based on the AP distance between contralateral ankle markers. The final score was estimated by taking the median of all the step lengths from the four laps.

(c) Average step time (ST_Av_, units: s)

This parameter was computed as the mean of the time between two consecutive initial contacts of alternative feet.

(d) Step length variability (SL_Vr_, units: %)

Step length variability was calculated as the root mean square of the SD of left step lengths and the SD of right step lengths and expressed as the mean step length percentage. The SD of left/right step length was estimated as $\frac{1}{1.35}$ times the interquartile range (IQR) of all the left/right step lengths collated from the four 6-s trials. IQR was used as the best estimator for SD to account for data skew resulting from a potential algorithm or signal anomaly [51].

(e) Step time variability (ST_Vr_, units: %)

Step time variability was calculated as the root mean square of the SD of left step times and the SD of right step times and expressed as the mean step time percentage.

(f) Step length asymmetry (SL_As_, units: %)

This parameter was computed as the percentage difference between left and right mean step lengths compared to the overall mean step length.

(g) Step time asymmetry (ST_As_, units: %)

This parameter was computed as the percentage difference between left and right mean step times compared to the overall mean step time.

(h) Walking speed (WS, units: m/s)

This parameter was computed as the mean of the ratios of step length to step time.

For the static balance task, the following outcome was selected to capture static postural control:

(a) Postural stability (PS, units: −ln[m/s^2^])

Postural stability was computed as the negative natural logarithm of the mean of the absolute acceleration along mediolateral, anterior–posterior, and vertical axes resultant vector. Postural stability was also computed separately for the mediolateral (PS_ML_) and the anterior–posterior axis (PS_AP_). As the negative natural logarithm was taken, high postural stability scores meant a low centre of mass accelerations and, thus, a good balance performance. For the 3D MoCap system, acceleration time series were constructed from displacement signals by double differentiation. A wavelet-based differentiation method was used to reduce the amplification of the sensor noise caused by numerical differentiation [50]. This parameter evaluated the participants’ stability through the analysis of the smartphone acceleration as an index of the participants’ centre of mass acceleration.

### 2.6. Statistical Analyses

#### 2.6.1. Validity of Gait and Balance Outcomes

To estimate concurrent validity of the G&B’s gait outcomes, the consistency and absolute agreement with the 3D MoCap system were calculated using the partial Pearson’s product–moment correlation coefficients and Bland–Altman limits of agreement (LoA), respectively. Data from the three test repetitions were included in the analysis. To account for repeated measures, a partial correlation coefficient (r_p_) was computed by first modelling the outcome from each instrument separately with a random-effects model, which included random intercepts for test repetitions, and then the Pearson’s product–moment correlation was obtained for the residuals from the two models [52]. The 95% LoA were calculated as the mean ± 1.96 × SD of the pairwise differences between the G&B application and MoCap outcomes. LoA included both the systematic and random errors, and thus, quantified the absolute agreement between the two systems. LoA were also calculated in percentage scores (LoA%) by dividing the absolute maximum of the 95% LoA by the mean of the outcomes across the two systems.

For concurrent validity of postural stability during the static balance tasks, the consistency between the two systems was evaluated with partial Pearson’s product–moment correlation, including data from all three test repetitions in the same model. A single task-level model was used as the postural stability scores obtained from the 3D MoCap system were affected by the high noise content caused by the double numerical differentiation of the displacement time series. Inclusion of all the tasks in the same model expanded the outcome variance owing to the presence of between-task differences. The low signal-to-noise ratio did not allow assessment of LoA between the two systems as a poor agreement could not be decisively ascribed to noise because of double numerical differentiation or sensor noise. To account for the repeated measures, a partial correlation coefficient (r_p_) was computed by first modelling the outcome from each instrument separately with a random-effects model, which included random intercepts for participants and separate random intercepts for test repetitions, and then the Pearson’s product–moment correlation was obtained for the residuals from the two models.

#### 2.6.2. Reliability and Responsiveness of Gait and Balance Outcomes

The within-session relative reliability of each outcome was assessed using two-way random-effects models to estimate the intra-class correlation coefficient (ICC_2,1_) for absolute agreement between single measures [53,54,55] using data from the three test repetitions (ICC^All^) and the data from repetitions 2 and 3 (ICC^2−3^). ICC^All^ was calculated to provide a conservative reliability index for those cases in which only one test could be performed. ICC^2−3^ provided a reliability index reducing the influence of the repetition effects. In addition, the standard error of measurement (SEM) was used to quantify the within-session absolute reliability. The SEM was computed as the standard deviation of the residuals taken from the ICC^All^ model. The SEM was interpreted as the error associated with a single measurement taken on a random day. The SEM was also expressed as a percentage of the repetition 3 mean (SEM%) to facilitate its interpretation. The mean of the third repetition was used for the SEM% calculation to reduce the influence of the repetition effects.

Responsiveness of the outcomes to repetition effects across test repetitions within a single task and to differences across tasks was evaluated using separate one-way repeated measure ANOVAs with sphericity correction [56]. For differences across tasks, data from the third repetition were used. Pairwise comparisons were performed with repeated measure t tests along with the false discovery rate correction. These ANOVAs evaluated the sensitivity of the gait and balance outcomes to subtle performance changes across repeated attempts of the same task and to differences across tasks.

#### 2.6.3. Assumptions, Data Presentation and Interpretation

Descriptive results were presented as mean ± standard deviation. The number of decimal places for each outcome was chosen such that the SEM for the outcome had one significant digit [57]. For the sake of uniformity, means and standard deviations for all the static tasks were presented with two decimal digits to the right of the decimal point. Normality and homogeneity of variance assumptions for the model residuals were evaluated with QQ-plots and fitted-vs.-residuals plots. Statistical significance was set at 0.05. Consistency interpretation was based on the lower bound for the 95% CI of r_p_. Absolute agreement interpretation was based on the LoA%. Specifically, correlation coefficients and LoA% were interpreted as follows [26]: excellent (>0.900, 0.0–4.9%), good (0.750–0.899, 5.0–9.9%), moderate (0.500–0.749, 10.0–49.9%) and poor (<0.500, >50.0%). Reliability interpretation was based on the lower bound for the 95% confidence interval of the ICCs as follows [58]: excellent (0.90–1), high (0.7–0.899), moderate (0.50–0.699), and poor (0–0.499).

## 3. Results

### 3.1. Validity of Gait and Balance Outcomes

Validity statistics for the gait outcomes are listed in Table 2. Average step time from both the tasks showed excellent consistency and agreement scores (r_p_ = 0.98; LoA% = 3%), while average step length and walking speed showed moderate-to-good scores (0.70 ≤ r_p_ ≤ 0.86; 10% ≤ LoA% ≤ 12%). The periodicity index also showed moderate-to-good consistency and agreement between systems for both tasks (0.55 ≤ r_p_ ≤ 0.67; 8% ≤ LoA% ≤ 10%). The variability and asymmetry measures for step length and time had poor consistency and agreement scores (r_p_ ≤ 0.34; LoA% ≥50%).

Task-level consistency statistics for postural stability outcomes from the static tasks are listed in Table 3. Postural stability computed from the three axes showed good consistency, postural stability along the mediolateral axis showed moderate consistency and postural stability along the anterior–posterior axis showed excellent consistency.

### 3.2. Reliability and Responsiveness of Gait and Balance Outcomes

Reliability and responsiveness statistics for the gait outcomes are shown in Table 4. Based on the lower bound scores, periodicity index showed high reliability scores in both gait tasks (0.71 ≤ ICC^All^ ≤ 0.75; 0.75 ≤ ICC^2−3^ ≤ 0.79; SEM% = 1%). Average step length and step time as well as walking speed showed moderate-to-high reliability scores across the two tasks (0.63 ≤ ICC^All^ ≤ 0.87; 0.82 ≤ ICC^2−3^ ≤ 0.92; 2% ≤ SEM% ≤ 4%). The variability and asymmetry measures for step length and time generally showed poor reliability scores. However, step length asymmetry showed moderate relative reliability scores across both tasks when the repetition effect was removed (0.53 ≤ ICC^2−3^ ≤ 0.62). Regarding the repetition effect on repeated gait tasks, statistically significant (*p* < 0.05) changes across the three test repetitions were detected during the head turning walking task (WT_HT_) for all variables except step time asymmetry. For the walking task with head facing forward (WT_HF_), the only apparent significant changes across test repetitions were found in average step time, step time variability and walking speed. Between-task comparisons showed significantly (*p* < 0.05) reduced periodicity, step length, and walking speed, and longer step time, during walking with head turns compared to walking with head forward.

**Figure 4:**
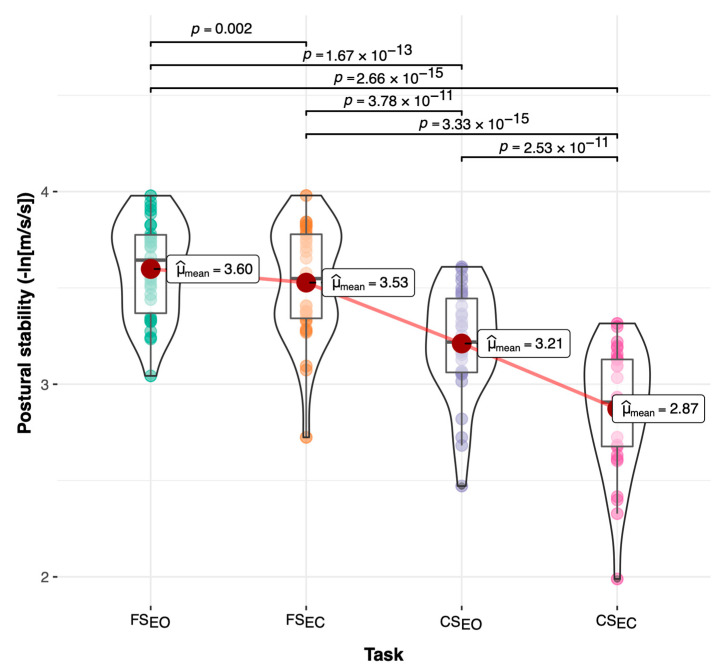
Pairwise comparisons for postural stability across static tasks. FS_EO_ = static balance task on a firm surface with eyes opened; FS_EC_ = static balance task on a firm surface with eyes closed (decreased visual feedback); CS_EO_ = static balance task on compliant surface with eyes opened (altered proprioceptive feedback); CS_EC_ = static balance task on compliant surface with eyes closed (decreased visual and proprioceptive feedback).

Reliability and responsiveness statistics for the postural stability outcomes from the static tasks are shown in Table 5. The postural stability (PS) outcome showed high reliability scores (0.71 ≤ ICC^2−3^ ≤ 0.86; SEM% = 3%) when the repetition effect was removed for all the tasks except CS_EC,_ which showed moderate reliability (ICC^2−3^ = 0.52; SEM% = 6%). In addition, postural stability on the anterior–posterior axis showed poorer reliability scores (0.38 ≤ ICC^2−3^ ≤ 0.78; 2% ≤ SEM% ≤ 4%) than the mediolateral axis (0.53 ≤ ICC^2−3^ ≤ 0.79; 3% ≤ SEM% ≤ 4%). Finally, SEM scores during the compliant eyes-closed task (CS_EC_) were larger (4% ≤ SEM% ≤ 6%) compared to the other tasks (2% ≤ SEM% ≤ 3%). Regarding the effects of repeated postural stability tests, statistically significant changes (*p* < 0.05) over time were detected in at least one of the outcomes for all tasks except for the compliant eye-closed task (CS_EC_). Between-task comparisons showed significant differences between the four static tasks. Pairwise comparisons across the tasks illustrated in Figure 4 showed a significant postural stability reduction (*p* < 0.01) across the tasks caused by the reduced visual and/or proprioceptive feedback. These significant pairwise differences demonstrated the ability of the postural stability outcome included in the application to detect reduction in the balance performance with increased task difficulty under compliant surface and closed eyes conditions.

## 4. Discussion

Overall, the main results of this study confirmed that a novel smartphone application measuring both postural stability and spatiotemporal gait characteristics is reliable and valid for selected but not all outcomes.

### 4.1. Validity of Gait and Balance Outcomes Obtained from the Smartphone Application

Regarding gait outcomes, similar to previous findings reported by Silsupadol and colleagues [34,59], spatiotemporal gait parameters such as average step time, average step length, and walking speed showed moderate-to-excellent consistency and absolute agreement. These results reinforced that, through appropriate algorithms, inertial sensors embedded in current smartphones can provide results as valid as those displayed by inertial measurement units typically designed for research [25]. Notably, the periodicity index also showed moderate-to-good validity; although, for the 3D MoCap system, this index was based on a double differentiated displacement signal, which typically amplifies the high-frequency noise [50]. Validity of the periodicity index has important implications for gait assessment in pathological populations as it is sensitive to both asymmetry and within-stride variability [44,49,60,61,62,63,64,65].

While assessing the validity of some spatiotemporal gait parameters was successful, this was not the case for step length/time variability and for step length/time asymmetry. These poor results verified the difficulty of capturing step-to-step variations using a single sensor placed on the low back, even though the gait tasks were performed in a straight line and not in a free-living environment [59]. A possible explanation for these results is the signal noise caused by the integration procedure applied as part of the inverted pendulum gait model used for obtaining these parameters from acceleration time series [43]. Analysis of gait data with neural networks may lead to more promising results [66].

Finally, the postural stability outcomes from the four static tasks demonstrated good-to-excellent validity between the smartphone app and the 3D MoCap system. It must be pointed out that the agreement analysis could not be performed because the acceleration signal constructed from the displacement signal of the 3D MoCap system was influenced by noise induced by the numerical differentiation [50]. Nevertheless, the consistency analysis demonstrated that both systems similarly classified participants according to their static balance performance.

### 4.2. Reliability of Gait and Balance Outcomes Obtained from the Smartphone Application

Besides their validity, the periodicity index, average step time, average step length, and walking speed obtained from the G&B app showed high relative reliability, especially when the repetition effect was removed. These results confirmed that gait parameters obtained from an inertial sensor placed on the lower trunk could be used for classifying healthy individuals according to their gait performance. These parameters also showed relatively low SEM values ranging from 1 to 4%. This low variability suggested that these parameters may be stable enough to detect subtle but real longitudinal changes in gait performance, distinguishing them from the natural within-subject variability [67]. However, further research should determine the natural variation in these parameters across different sessions rather than relying on these within-session findings.

In this sense, as one of the aims of this study was to assess the smartphone ability to detect subtle changes in gait and balance performance, we assessed the responsiveness of the different parameters by comparing different tasks and analysing the potential repetition effect. As Table 4 shows, the periodicity and the spatial-temporal gait outcomes could discriminate between the different gait tasks. It must be underlined that differences between gait tasks were subtle but still higher than most of the SEM scores observed in the reliability analysis. Spatiotemporal parameters also captured within-session changes caused by the task repetition mainly observed when participants walked while turning their heads.

Step length/time variability and asymmetry parameters generally showed poor reliability, likely caused by the low number of steps analysed, much lower than the 50 minimum steps proposed by Galna et al. [68] during continuous walking tasks. Although a previous study suggested that 16 strides could be enough to reliably estimate the step time variability during continuous walking [69], we could not replicate these results in our discontinuous gait tasks designed for short corridors. Nonetheless, the step length asymmetry showed moderate reliability in both gait tasks when the repetition effect was removed. This is a promising finding and needs to be investigated further in pathological populations expected to have a significantly asymmetric gait [63]. From the authors’ point of view, the moderate reliability of step length asymmetry combined with the high reliability of the periodicity index supported the hypothesis that the low back acceleration profile during walking may be used to capture meaningful information related to the participants’ gait variability and asymmetry.

Regarding the reliability analysis of the postural stability during the static balance tasks, our results confirmed that the selected balance tasks were able to consistently rank and assign the same scores across test repetitions to participants according to their balance performance [24]. In addition, SEM scores (2% < SEM < 6%) were similar to those found using inertial sensors in lab settings in the same balance tasks [70,71]. This low within-session variability suggested that the smartphone app may have sufficient stability to detect small but real changes in balance performance. In line with this, postural stability outcomes were sensitive enough to reveal balance impartments induced by more challenging task conditions [70] and small improvements driven by the task repetitions. These results reinforced the potential usefulness of the smartphone-based assessment to detect small changes in balance performance over the course of a rehabilitation or training program.

### 4.3. Clinical Implications

The availability of affordable yet reliable and valid solutions for clinically meaningful gait and balance assessment has important implications for health and disability outcomes. On the one hand, it might facilitate tracking the effectiveness of rehabilitation or training processes. On the other hand, it could help identify the underlying causes of balance and gait impairments, enhancing clinical decision making. In this sense, clinical decisions could also benefit from future studies on other populations that obtain reference scores for the gait and balance outcomes. This could enable the prediction of adverse events such as the risk of falling or the progress in sensorimotor impairment associated with neurodegenerative diseases. Finally, one of the most promising applications is the instant feedback obtained from the smartphone app to control the challenge imposed by the rehabilitation exercises (i.e., training intensity).

### 4.4. Limitations

The current study has some limitations. First, this study has shown the validity and reliability of the smartphone app in a wide range of ages; the focus was on healthy individuals without any identifiable balance impairment. Futures studies on populations with gait and balance impairments are needed to learn the boundaries of the algorithms for gait parameter estimation. In this sense, the accuracy of the gait outcomes might be compromised by several factors such as slower gait speeds, short strides, or significant gait asymmetries, which are easily observed in several pathological conditions such as stroke, multiple sclerosis, and vestibular disorders [72].

## 5. Conclusions

This study reinforced the emerging evidence that an embedded inertial sensor-based smartphone application can provide a valid and reliable estimation of several gait and balance parameters in healthy adults. During straight line walking and walking with side-to-side head turns, step length, step time, walking speed, and periodicity index were both reliable and valid compared to a 3D motion capture system. Variability and asymmetry of step length and step time were generally neither reliable nor valid. During static balance tasks, postural stability measures were found to be reliable and valid compared to a 3D motion capture system. Gait and balance outcomes were also sensitive to subtle performance changes across repetitions of the same task and to performance differences between tasks. These findings are limited to a healthy population. Nonetheless, these findings provide a solid foundation for future investigations of the proposed application and its structured protocol to assess gait and balance in people with vestibular disorders, multiple sclerosis, Parkinson’s disease, and older adults at risk of falls.

## Figures and Tables

**Table 1:** Participant characteristics.

Participant No.	Sex	Age (Years)	Height (cm)	Mass (kg)
1	Female	37	152	50.9
2	Female	27	168	76.2
3	Male	25	180	87.3
4	Female	27	166.5	63
5	Male	39	164.5	63.9
6	Female	36	168.6	64.7
7	Male	28	186	84.2
8	Male	38	170	95.7
9	Male	53	177	76.7
10	Female	32	164.3	65.5
11	Male	27	176.6	71.1
12	Male	57	179.5	83.4
13	Female	28	159.4	83.2
14	Male	47	178.5	81.3
15	Male	48	174.5	75.3
16	Female	41	162	68.8
17	Female	43	161	54.4
18	Female	59	163	57.5
19	Female	59	155	67.4
20	Male	30	181.5	85.2
21	Male	46	175.5	71.1
22	Male	51	180	78.3
23	Female	46	163	62.4
24	Female	68	159	65
25	Female	52	157	66
26	Male	61	166	86.4
27	Male	67	183	104.4
28	Male	69	178	104.1
29	Female	60	152	81.5
30	Female	63	145	54.1

**Table 2:** Validity of gait outcomes obtained from the Gait&Balance (G&B) application compared to the 3D motion capture (MoCap) system.

Outcome	MoCap	G&B App	95% LoA	LoA%	Agreement Interpretation	r_p_ [95% CI]	Consistency Interpretation
Comfortable walking with the head forward							
Periodicity (%)	68 ± 3	70 ± 3	−2, 7	10	Moderate	0.69 [0.55, 0.79]	Moderate
SL_Av_ (m)	0.68 ± 0.06	0.67 ± 0.06	−0.07, 0.07	11	Moderate	0.81 [0.72, 0.87]	Moderate
ST_Av_ (s)	0.54 ± 0.04	0.54 ± 0.04	−0.01, 0.02	3	Excellent	0.99 [0.98, 0.99]	Excellent
SL_Vr_ (%)	4 ± 1	3 ± 1	−4, 2	109	Poor	0.20 [−0.02, 0.39]	Poor
ST_Vr_ (%)	2.5 ± 0.8	2.9 ± 1	−1.5, 2.2	80	Poor	0.40 [0.20, 0.57]	Poor
SL_As_ (%)	4 ± 3	3 ± 2	−8, 5	217	Poor	0.37 [0.17, 0.54]	Poor
ST_As_ (%)	2 ± 2	3 ± 2	−3, 6	212	Poor	0.37 [0.17, 0.54]	Poor
WS (m/s)	1.27 ± 0.16	1.25 ± 0.14	−0.15, 0.12	12	Moderate	0.91 [0.86, 0.94]	Good
Comfortable walking while turning the head							
Periodicity (%)	67 ± 3	69 ± 3	−2, 6	8	Good	0.77 [0.67, 0.84]	Moderate
SL_Av_ (m)	0.64 ± 0.05	0.65 ± 0.05	−0.06, 0.07	10	Moderate	0.80 [0.70, 0.86]	Moderate
ST_Av_ (s)	0.55 ± 0.04	0.56 ± 0.04	−0.01, 0.02	3	Excellent	0.99 [0.98, 0.99]	Excellent
SL_Vr_ (%)	5 ± 2	3 ± 1	−6, 3	140	Poor	0.37 [0.17, 0.54]	Poor
ST_Vr_ (%)	2.5 ± 0.9	3.0 ± 0.8	−1.3, 2.2	79	Poor	0.44 [0.24, 0.59]	Poor
SL_As_ (%)	5 ± 4	3 ± 3	−8, 5	197	Poor	0.52 [0.34, 0.66]	Poor
ST_As_ (%)	2 ± 2	3 ± 2	−4, 7	283	Poor	0.14 [−0.07, 0.34]	Poor
WS (m/s)	1.18 ± 0.13	1.16 ± 0.13	−0.13, 0.11	11	Moderate	0.88 [0.82, 0.92]	Good

Descriptive statistics are presented as mean ± standard deviation. LoA = limits of agreement; LoA% = upper limits of agreement in percentage; ‘r_p_’ = partial Pearson’s product–moment correlation coefficient; CI = confidence intervals; SL_Av_ = average step length; ST_Av_ = average step time; SL_Vr_ = step length variability; ST_Vr_ = step time variability; SL_As_ = step length asymmetry; ST_As_ = step time asymmetry; WS = walking speed.

**Table 3:** Validity of postural stability outcomes obtained from the Gait&Balance (G&B) application compared to the 3D motion capture (MoCap) system.

Outcome	r_p_ [95% CI]	Interpretation
PS	0.87 [0.84, 0.89]	Good
PS_ML_	0.73 [0.68, 0.78]	Moderate
PS_AP_	0.95 [0.93, 0.96]	Excellent

‘r_p_’ = partial Pearson’s product–moment correlation coefficient; CI = confidence intervals; PS = postural stability (units: −ln[m/s/s]); PSML = postural stability in the mediolateral axis (units: −ln[m/s/s]); PSAP = postural stability in the anterior–posterior axis (units: −ln[m/s/s]).

**Table 4:** Reliability, repetition effect, and between-task comparisons of the gait outcomes obtained from the Gait&Balance (G&B) application.

Outcome	Task	Test 1, Test 2, Test 3	*F, p*	SEM, SEM%	ICC^All^ [95% CI], Interpretation	ICC^2−3^ [95% CI] Interpretation
Periodicity (%)	WT_HF_	70 ± 3, 70 ± 3, 71 ± 2	2.48, 0.1	1, 1	0.85 [0.75, 0.92], ℍ	0.90 [0.79, 0.95], ℍ
	WT_HT_	68 ± 3, 69 ± 3, 70 ± 3	8.64, 0.001	1, 1	0.84 [0.71, 0.92], ℍ	0.88 [0.75, 0.94], ℍ
	Between-task ANOVA		20.23, <0.001			
SL_Av_ (m)	WT_HF_	0.67 ± 0.06, 0.67 ± 0.06, 0.68 ± 0.05	2.78, 0.1	0.01, 2	0.93 [0.87, 0.96], ℍ	0.96 [0.92, 0.98], 𝔼
	WT_HT_	0.64 ± 0.05, 0.65 ± 0.05, 0.65 ± 0.05	16.72, <0.001	0.01, 2	0.91 [0.77, 0.96], ℍ	0.96 [0.85, 0.98], 𝔼
	Between-task ANOVA		40.22, <0.001			
ST_Av_ (s)	WT_HF_	0.55 ± 0.04, 0.54 ± 0.04, 0.53 ± 0.04	6.72, 0.008	0.01, 3	0.85 [0.73, 0.92], ℍ	0.95 [0.88, 0.97], ℍ
	WT_HT_	0.57 ± 0.05, 0.56 ± 0.05, 0.55 ± 0.04	10.59, <0.001	0.01, 2	0.90 [0.80, 0.95], ℍ	0.96 [0.91, 0.98], 𝔼
	Between-task ANOVA		9.34, 0.005			
SL_Vr_ (%)	WT_HF_	3.2 ± 0.8, 2.9 ± 0.8, 3.1 ± 0.8	2.1, 0.1	0.6, 19	0.48 [0.26, 0.68], ℙ	0.53 [0.23, 0.75], ℙ
	WT_HT_	3.9 ± 1.1, 3.4 ± 0.9, 3.2 ± 0.7	6.06, 0.006	0.8, 26	0.16 [−0.30, 0.39], ℙ	0.32 [−0.3, 0.60], ℙ
	Between-task ANOVA		0.15, 0.7			
ST_Vr_ (%)	WT_HF_	3.2 ± 1.1, 2.8 ± 1, 2.6 ± 0.8	3.79, 0.030	0.9, 33	0.14 [−0.50, 0.38], ℙ	0.40 [−0.32, 0.40], ℙ
	WT_HT_	3.3 ± 0.9, 2.8 ± 0.7, 2.9 ± 0.8	3.24, 0.049	0.7, 25	0.23 [0.20., 0.47], ℙ	0.20 [−0.17, 0.52], ℙ
	Between-task ANOVA		3.18, 0.090			
SL_As_ (%)	WT_HF_	3 ± 2, 3 ± 3, 3 ± 2	0.88, 0.400	1, 34	0.79 [0.66, 0.88], 𝕄	0.80 [0.62, 0.90], 𝕄
	WT_HT_	4 ± 3, 3 ± 2, 3 ± 2	3.54, 0.044	2, 54	0.61 [0.42, 0.78], ℙ	0.74 [0.53, 0.87], 𝕄
	Between-task ANOVA		1.22, 0.279			
ST_As_ (%)	WT_HF_	4 ± 2, 3 ± 2, 3 ± 2	1.54, 0.227	1, 44	0.58 [0.38, 0.75], ℙ	0.71 [0.47, 0.85], ℙ
	WT_HT_	3 ± 3, 3 ± 2, 3 ± 2	0.37, 0.686	2, 50	0.61 [0.41, 0.77], ℙ	0.66 [0.39, 0.82], ℙ
	Between-task ANOVA		0.29, 0.593			
WS (m/s)	WT_HF_	1.23 ± 0.15, 1.26 ± 0.13, 1.27 ± 0.13	5.73, 0.014	0.05, 4	0.85 [0.73, 0.92], ℍ	0.92 [0.85, 0.96], ℍ
	WT_HT_	1.13 ± 0.13, 1.17 ± 0.13, 1.19 ± 0.12	16.14, <0.001	0.04, 4	0.83 [0.63, 0.92], 𝕄	0.93 [0.82, 0.97], ℍ
	Between-task ANOVA		35.57, <0.001			

Descriptive statistics are presented as mean ± standard deviation. SEM = standard error of measurement expressed in the outcome units; SEM% = standard error of measurement expressed in percentage with respect to the outcome mean; ICC^All^ = intraclass correlation coefficient for absolute agreement between single measures using data from the three tests; ICC^2−3^ = intraclass correlation coefficient for absolute agreement between single measures using data from the last two tests; CI = confidence intervals; WT_HF_ = Comfortable walking with the head forward; WT_HT_ = Comfortable walking while turning the head; SL_Av_ = average step length; ST_Av_ = average step time; SL_Vr_ = step length variability; ST_Vr_ = step time variability; SL_As_ = step length asymmetry; ST_As_ = step time asymmetry; WS = walking speed; 𝔼 = excellent; ℍ = high; 𝕄 = moderate; ℙ = poor.

**Table 5:** Reliability, repetition effect, and between-task comparisons of the postural stability outcomes obtained from the Gait&Balance (G&B) application during the static balance tasks.

Outcome	Task	Test 1, Test 2, Test 3	*F, p*	SEM, SEM%	ICC^All^ [95% CI], Interpretation	ICC^2−3^ [95% CI], Interpretation
PS	FS_EO_	3.57 ± 0.26, 3.54 ± 0.26, 3.6 ± 0.25	2.20, 0.1	0.1, 3	0.84 [0.73, 0.91], ℍ	0.86 [0.71, 0.94], ℍ
	FS_EC_	3.47 ± 0.32, 3.48 ± 0.29, 3.53 ± 0.29	3.29, 0.048	0.09, 3	0.91 [0.84, 0.95], ℍ	0.92 [0.83, 0.96], ℍ
	CS_EO_	3.06 ± 0.27, 3.17 ± 0.32, 3.21 ± 0.28	20.83, <0.001	0.1, 3	0.83 [0.60, 0.93], 𝕄	0.93 [0.86, 0.97], ℍ
	CS_EC_	2.84 ± 0.34, 2.83 ± 0.26, 2.87 ± 0.32	0.60, 0.6	0.2, 6	0.72 [0.56, 0.84], 𝕄	0.73 [0.52, 0.86], 𝕄
	Between-task ANOVA		189.73, <0.001			
PS_ML_	FS_EO_	4.24 ± 0.29, 4.21 ± 0.29, 4.29 ± 0.29	4.58, 0.015	0.1, 3	0.84 [0.73, 0.92], ℍ	0.84 [0.61, 0.93], 𝕄
	FS_EC_	4.15 ± 0.36, 4.17 ± 0.31, 4.24 ± 0.32	4.72, 0.019	0.1, 3	0.88 [0.79, 0.94], ℍ	0.91 [0.79, 0.96], ℍ
	CS_EO_	3.77 ± 0.27, 3.91 ± 0.33, 3.95 ± 0.30	16.97, <0.001	0.1, 3	0.77 [0.52, 0.89], 𝕄	0.89 [0.79, 0.95], ℍ
	CS_EC_	3.64 ± 0.34, 3.63 ± 0.30, 3.67 ± 0.34	0.61, 0.5	0.2, 4	0.75 [0.60, 0.86], 𝕄	0.74 [0.53, 0.87], 𝕄
	Between-task ANOVA		110.53, <0.001			
PS_AP_	FS_EO_	4.19 ± 0.22, 4.16 ± 0.20, 4.2 ± 0.22	0.66, 0.5	0.1, 3	0.71 [0.54, 0.84], 𝕄	0.78 [0.59, 0.89], 𝕄
	FS_EC_	4.06 ± 0.28, 4.06 ± 0.25, 4.1 ± 0.27	1.46, 0.2	0.1, 2	0.87 [0.78, 0.93], ℍ	0.88 [0.77, 0.94], ℍ
	CS_EO_	3.72 ± 0.21, 3.81 ± 0.23, 3.83 ± 0.23	10.15, 0.001	0.1, 3	0.77 [0.58, 0.88], 𝕄	0.89 [0.78, 0.94], ℍ
	CS_EC_	3.45 ± 0.27, 3.43 ± 0.21, 3.48 ± 0.25	0.76, 0.5	0.1, 4	0.67 [0.49, 0.81], ℙ	0.65 [0.38, 0.81], ℙ
	Between-task ANOVA		187.82, <0.001			

Descriptive statistics are presented as mean ± standard deviation. SEM = standard error of measurement expressed in the outcome units; SEM% = standard error of measurement expressed in percentage with respect to the outcome mean; ICC^All^ = intraclass correlation coefficient for absolute agreement between single measures using data from the three tests; ICC^2−3^ = intraclass correlation coefficient for absolute agreement between single measures using data from the last two tests; CI = confidence intervals; PS = postural stability (units: −ln[m/s/s]); PS_ML_ = postural stability in the medial–lateral axis (units: −ln[m/s/s]); PS_AP_ = postural stability in the anterior–posterior axis (units: −ln[m/s/s]); FS_EO_ = static balance task on a firm surface with eyes opened; FS_EC_ = static balance task on a firm surface with eyes closed (decreased visual feedback); CS_EO_ = static balance task on compliant surface with eyes opened (altered proprioceptive feedback); CS_EC_ = static balance task on compliant surface with eyes closed (decreased visual and proprioceptive feedback); 𝔼 = excellent; ℍ = high; 𝕄 = moderate; ℙ = poor. Note: higher PS scores mean better balance.

## Data Availability

The data presented in this study are available on request from the corresponding author. The request will be forwarded to the university’s ethics committee which will decide access to data for the requested purpose.
